# Comparative absorption, distribution, and excretion of titanium dioxide and zinc oxide nanoparticles after repeated oral administration

**DOI:** 10.1186/1743-8977-10-9

**Published:** 2013-03-26

**Authors:** Wan-Seob Cho, Byeong-Cheol Kang, Jong Kwon Lee, Jayoung Jeong, Jeong-Hwan Che, Seung Hyeok Seok

**Affiliations:** 1 Department of Medicinal Biotechnology, College of Natural Resources and Life Science, Dong-A University, Busan, 604-714, Republic of Korea; 2 Biomedical Research Institute, Seoul National University Hospital, Seoul, 110-744, Republic of Korea; 3 Department of Toxicological Research, National Institute of Food and Drug Safety Evaluation, Korea Food and Drug Administration, Osong, 363-700, Republic of Korea; 4 Department of Microbiology and Immunology, and Institute of Endemic Disease, Seoul National University College of Medicine, Seoul, 110-799, Republic of Korea

**Keywords:** TiO_2_, ZnO, Oral administration, Absorption, Distribution, Excretion

## Abstract

**Background:**

The *in vivo* kinetics of nanoparticles is an essential to understand the hazard of nanoparticles. Here, the absorption, distribution, and excretion patterns of titanium dioxide (TiO_2_) and zinc oxide (ZnO) nanoparticles following oral administration were evaluated.

**Methods:**

Nanoparticles were orally administered to rats for 13 weeks (7 days/week). Samples of blood, tissues (liver, kidneys, spleen, and brain), urine, and feces were obtained at necropsy. The level of Ti or Zn in each sample was measured using inductively coupled plasma-mass spectrometry.

**Results:**

TiO_2_ nanoparticles had extremely low absorption, while ZnO nanoparticles had higher absorption and a clear dose-response curve. Tissue distribution data showed that TiO_2_ nanoparticles were not significantly increased in sampled organs, even in the group receiving the highest dose (1041.5 mg/kg body weight). In contrast, Zn concentrations in the liver and kidney were significantly increased compared with the vehicle control. ZnO nanoparticles in the spleen and brain were minimally increased. Ti concentrations were not significantly increased in the urine, while Zn levels were significantly increased in the urine, again with a clear dose-response curve. Very high concentrations of Ti were detected in the feces, while much less Zn was detected in the feces.

**Conclusions:**

Compared with TiO_2_ nanoparticles, ZnO nanoparticles demonstrated higher absorption and more extensive organ distribution when administered orally. The higher absorption of ZnO than TiO_2_ nanoparticles might be due to the higher dissolution rate in acidic gastric fluid, although more thorough studies are needed.

## Background

Nanomaterials have been developed for the food industry for uses such as food storage, preventing microbial growth, and as a nutritional ingredient [1,2]. Titanium dioxide (TiO_2_) and zinc oxide (ZnO) have unique physicochemical properties including a bright white color, ability to block UV light, and antimicrobial activity. TiO_2_ nanoparticles naturally exist in three different crystalline structures (anatase, rutile and brookite) and have a high refractive index. TiO_2_ is widely used as a pigment (paint, plastics, and paper), in personal care products (sunscreen and toothpastes), and in food (cream) [3]. The bright white color of micron-sized TiO_2_ might be more useful for some applications than nanosized TiO_2_ because TiO_2_ becomes more transparent as its particle size decreases. However, smaller TiO_2_ particles have higher UV-blocking properties, which can be advantageous for food storage [4]. In addition, nanosized TiO_2_ prevents microbial growth [5]. Because of these properties, nanosized TiO_2_ might be popular in the food industry with further technological developments. ZnO is another commonly used particle with similar utility to TiO_2_. In addition, ZnO is used for its antimicrobial properties and in nutritional supplements such as multivitamins [6,7]. Compared with micron-sized ZnO, nanosized ZnO might have better UV-blocking and anti-microbial properties and higher bioavailability [8].

The increased use of these particles might increase the possibility of human exposure through various routes such as inhalation, ingestion, and skin contact. In addition to uses for humans, nanomaterials are used with livestock and in the environment. TiO_2_ nanoparticles are generally considered to have low toxicity regardless of their size and crystallinity [9]. However, recent *in vitro* studies have reported that TiO_2_ nanoparticles are genotoxic through the production of reactive oxygen species [10,11], although *in vitro* studies have limited predictive value for *in vivo* situations. In contrast to TiO_2_ nanoparticles, ZnO nanoparticles are well known to have high toxicity. One of the mechanisms of this toxicity is their ionization in biological fluids [12].

Combined with toxicity data, kinetics data can provide the actual concentration of nanoparticles as they interact with biological systems. However, compared with other routes of administration, kinetics data following the oral administration of nanoparticles is limited. Humans have a higher chance of being exposed to TiO_2_ and ZnO nanoparticles in food-related products than other nanoparticles. Therefore, further evaluation of the tissue distribution and absorption of these nanoparticles following oral administration is needed to provide valuable information for assessing the risk of these nanoparticles. We selected TiO_2_ and ZnO to evaluate nanoparticle absorption, tissue distribution, and excretion following 13 weeks of repeated oral administration.

## Results

### Physicochemical characterization of nanoparticles

**Figure 1:**
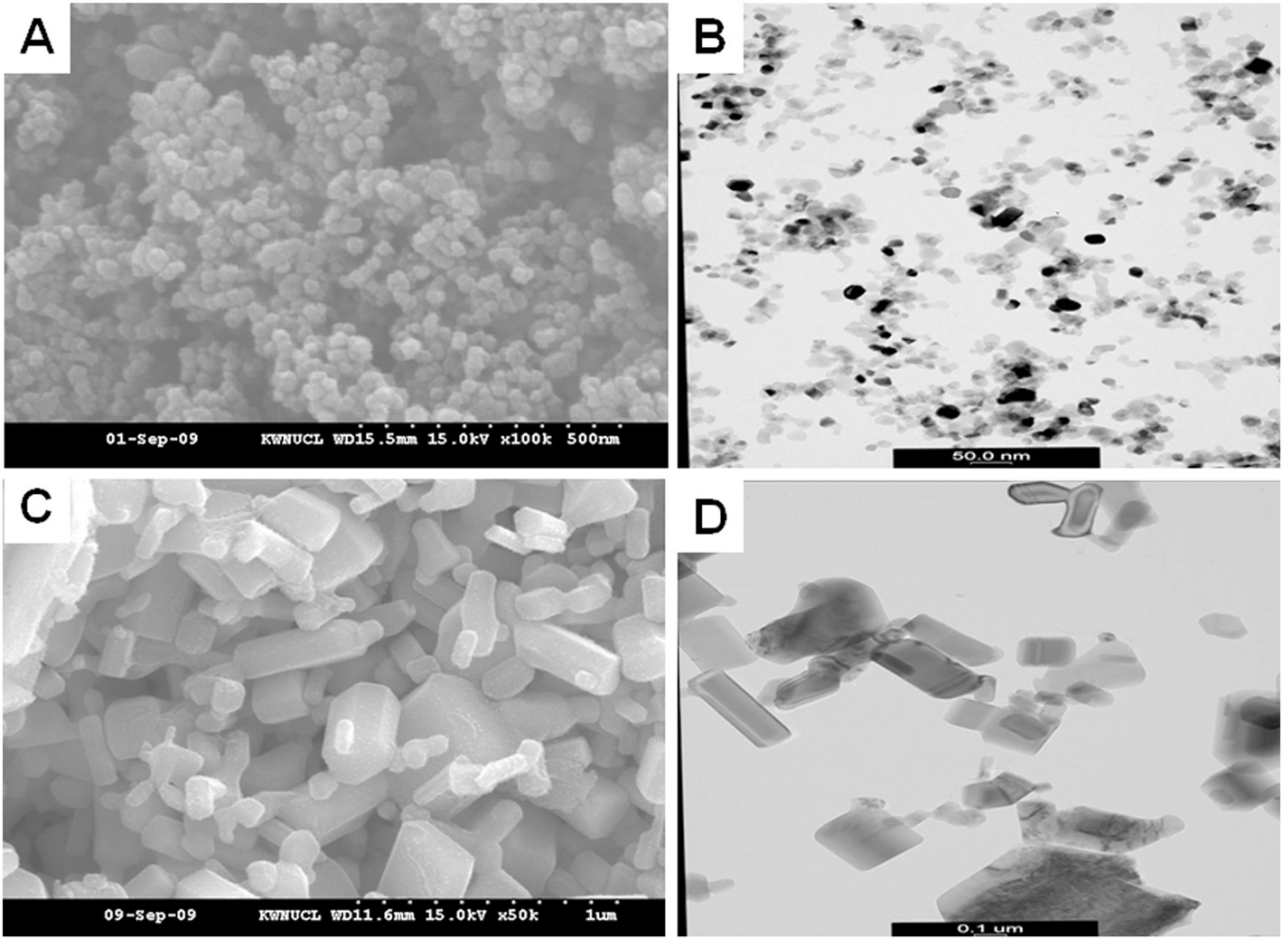
**SEM and TEM of TiO**_**2 **_**and ZnO nanoparticles.** SEM (**A**) and TEM (**B**) images of TiO_2_ nanoparticles show spherical shape. SEM (**C**) and TEM (**D**) images of ZnO nanoparticles show hexagonal shape.

TiO_2_ nanoparticles were spherical and ZnO nanoparticles were hexagonal (Figure 1). Table 1 summarizes the physicochemical properties of the TiO_2_ and ZnO nanoparticles. The primary sizes of TiO_2_ nanoparticles provided by the manufacturer were similar to those measured by scanning electron microscopy (SEM) and dynamic light scattering (DLS), while ZnO nanoparticles sizes differed significantly by the two measurement type. The hydrodynamic size of the ZnO nanoparticles suggested that ZnO formed small aggregates when dispersed in distilled water (DW). TiO_2_ nanoparticles had sizes similar to the primary particle size. No particles showed endotoxin contamination. The zeta potential of the TiO_2_ nanoparticles was 54.4 ± 0.7 mV for pH 2 and 7.9 ± 2.7 mV for pH 8. The zeta potential of ZnO was more negative than TiO_2_, at 11.7 ± 0.8 mV for pH 2 and -25.1 ± 2.6 mV for pH 8.

**Table 1:** Physicochemical characterization of metal oxide nanoparticles

**Particles**	**Crystallinity**	**Primary size (nm) measured by**		**Hydrodynamic size (nm)**^**a**^	**Surface area (m**^**2**^**/g)**^**b**^	**Endotoxin (pg/ml)**^**c**^
		**Manufacturer**	**SEM**			
TiO_2_	Anatase 80: 20 Rutile	21	26.4 ± 6.1	37.8 ± 0.4	50±15	N.D.
ZnO	Hexagonal	40	89.3 ± 44.7	201.8 ± 17.2	60±10	N.D.

*ND* not detectable. *SEM* scanning electron microscopy.

^a^Nanoparticles were dispersed in DW and measured by a dynamic light scattering method.

^b^Specific surface area by the Brunauer-Emmett-Teller method was provided by the manufacturer.

^c^Levels of endotoxin were measured using an Endpoint Chromogenic Limulus Amebocyte Lysate assay (Cambrex, MD, USA).

### Durability of nanoparticles under biological conditions

**Figure 2:**
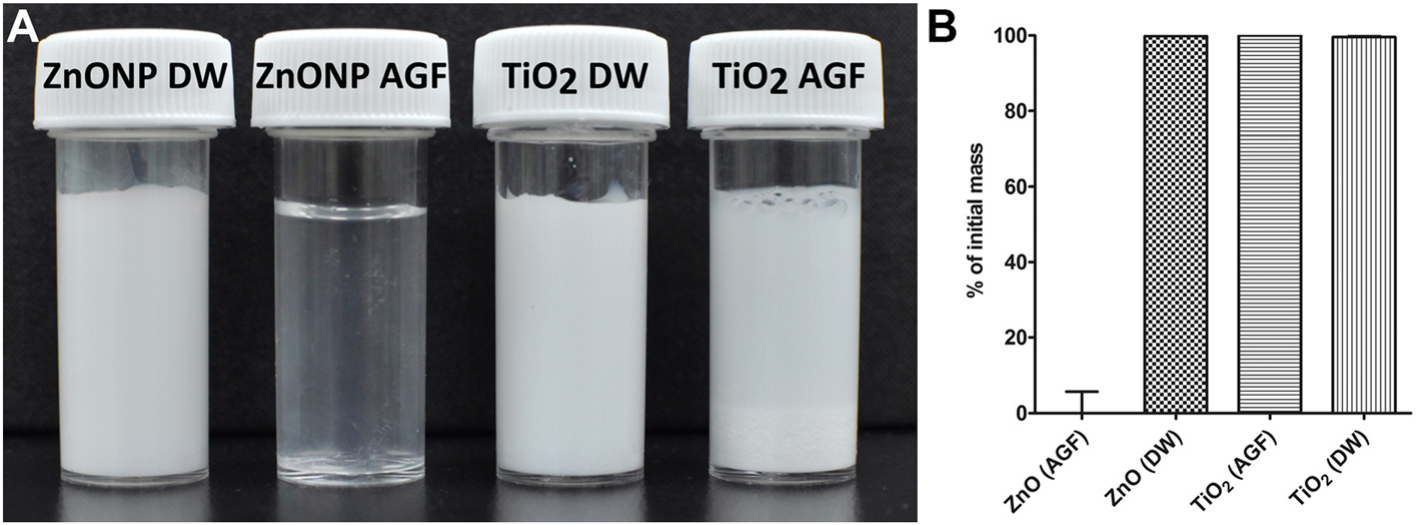
**Dissolution pattern of nanoparticles in the acidic gastric fluid. AGF, pH 1.5; basic condition, DW, pH 7.4.** Nanoparticles were incubated with AGF or DW for up to 24 h. (**A**) Image taken 5 min after incubation using a digital camera. (**B**) Percentage of dissolution measured using an ICP-MS at 24 h after incubation.

TiO_2_ nanoparticles showed minimal dissolution in both acidic gastric fluid (AGF) and pH 7.4 conditions (Figure 2A). However, ZnO nanoparticles dissolved in AGF within five minutes. In basic conditions, ZnO showed minimal dissolution after monitoring for up to 24 h. This dissolution pattern was confirmed by an inductively coupled plasma-mass spectrometry (ICP-MS) (Figure 2B).

### Body weight changes

When nanoparticles were administered for consecutive 13-week to female Sprague-Dawley rats, the body weight of given a high dose of ZnO nanoparticles was significantly lower than animals given in the vehicle control (Additional file 1: Tables S1–S4). However, other ZnO-treatment groups and the TiO_2_ nanoparticle-treatment groups showed no significant changes compared with the vehicle-control group.

### Absorption of nanoparticles

**Figure 3:**
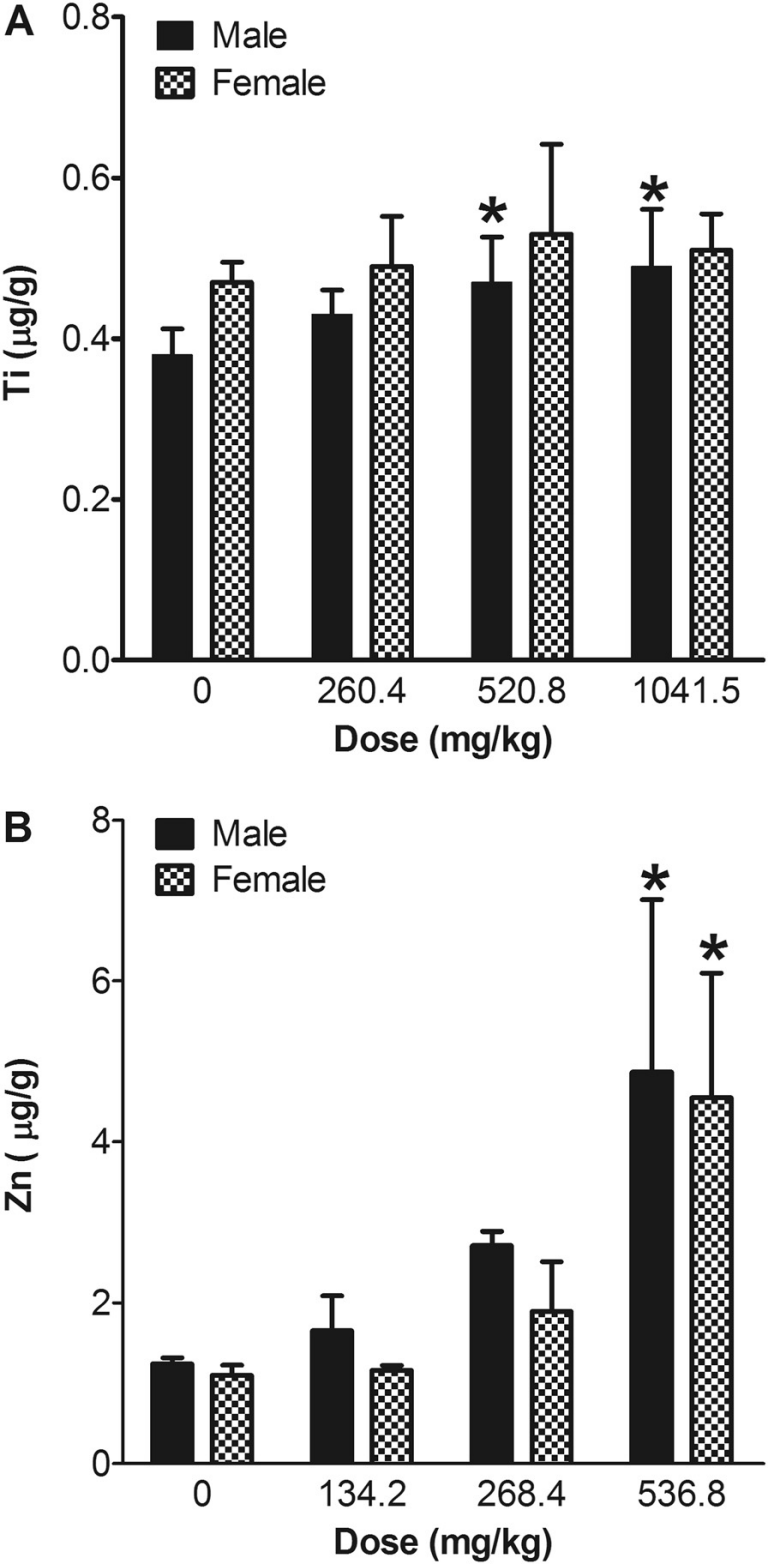
**Systemic absorption of nanoparticles from the gastrointestinal tract after 13 weeks of repeated oral treatment.** Ti concentration in whole blood (**A**) showed about a 10-fold lower absorption than Zn concentration in whole blood (**B**) in both sexes. Values are mean ± S.D. and *n* = 11. Significance versus vehicle control: ^*^p < 0.05.

The concentrations of Zn in the blood of the ZnO-treatment groups demonstrated a clear dose-response relationship (Figure 3). However, the concentrations of Ti in the male TiO_2_-treatment groups showed a less steep slope in the dose-response relationship. Female rats treated with TiO_2_ showed no dose-response relationship for blood concentration. The Zn blood concentration in the ZnO-treatment groups was almost 10-fold higher than the Ti concentration in the TiO_2_-treatment groups.

### Distribution of nanoparticles

**Figure 4:**
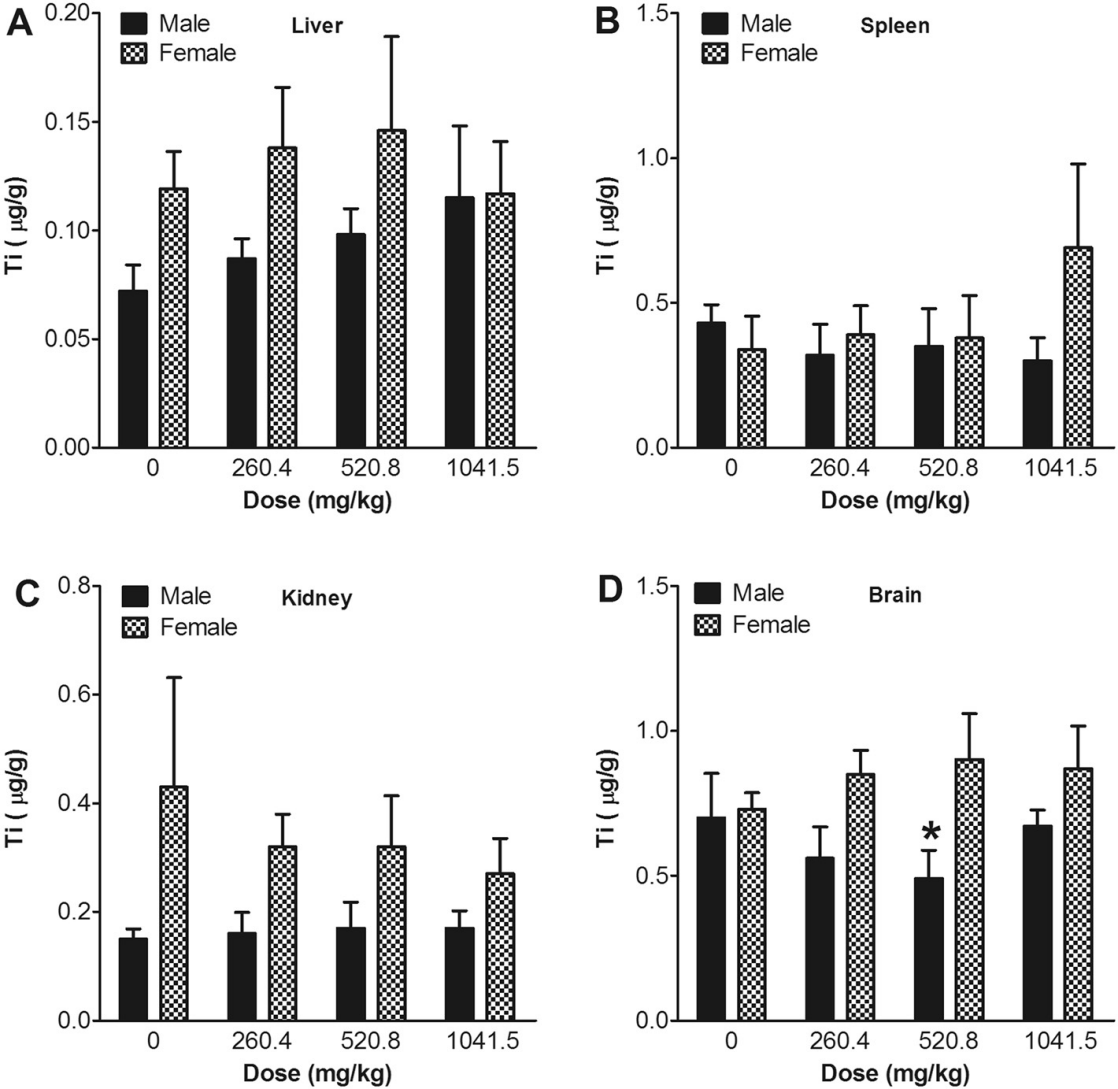
**Concentration of Ti in tissues after 13 weeks of consecutive oral administration of TiO**_**2 **_**nanoparticles.** The concentrations of Ti in the liver (**A**), spleen (**B**), kidney (**C**), and brain (**D**) were evaluated using an ICP-MS. Values are mean ± S.D. and *n* = 11. Significance versus vehicle control: ^*^p < 0.05.

**Figure 5:**
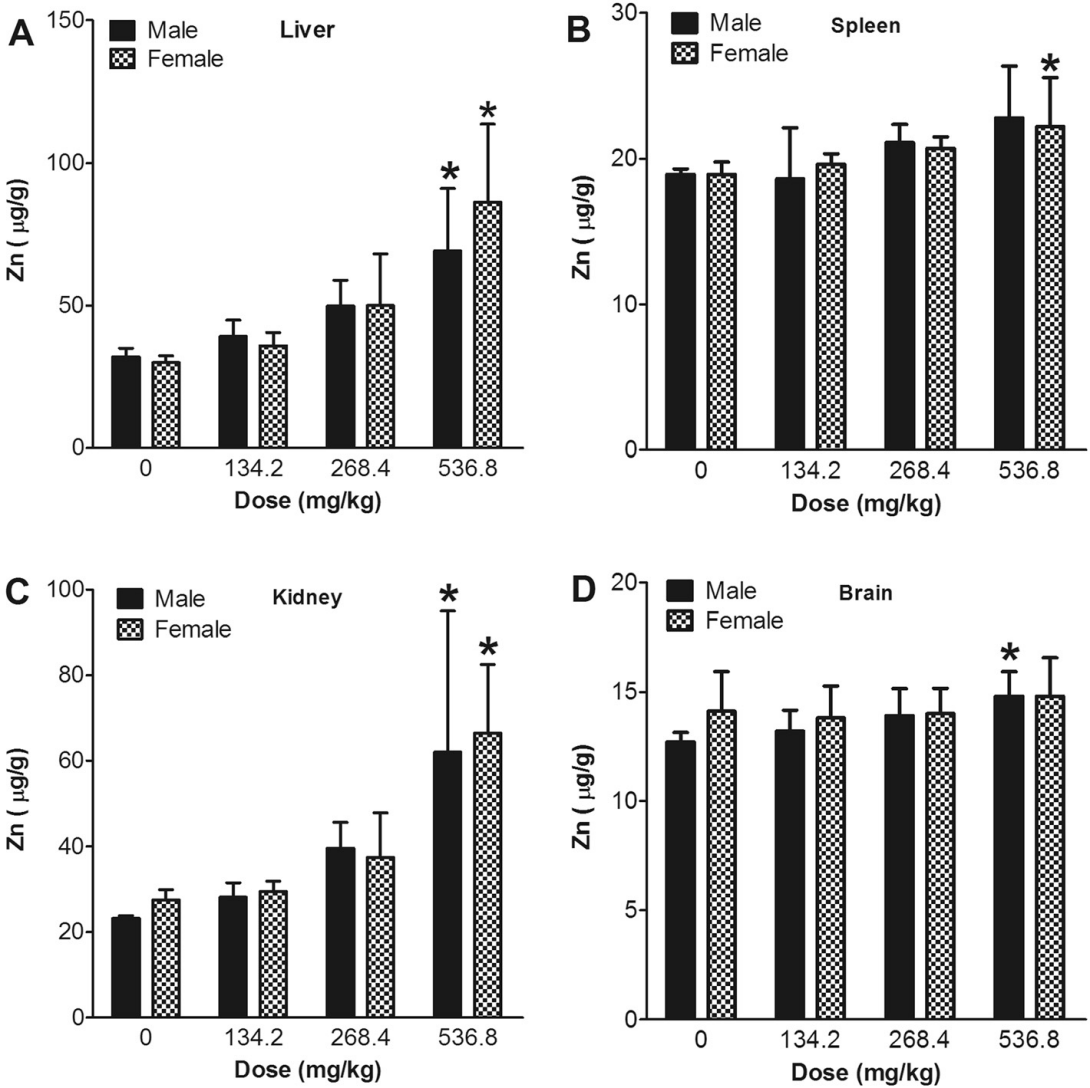
**Concentration of Zn in tissues after 13 weeks of consecutive oral administration of ZnO nanoparticles.** The concentrations of Zn in the liver (**A**), spleen (**B**), kidney (**C**), and brain (**D**) were evaluated using an ICP-MS. Values are mean ± S.D. and *n* = 11. Significance versus vehicle control: ^*^p < 0.05.

The distribution patterns of Ti and Zn in the liver, spleen, kidney, and brain after 13 weeks of repeated oral administration of TiO_2_ or ZnO nanoparticles were measured using ICP-MS. Ti concentrations in the TiO_2_-treatment groups showed no significant increase in the sampled organs compared with the vehicle control group (Figure 4 and Additional file 2: Figure S1). The Zn concentration in the liver and kidney showed a significant increase in the highest dose group with a positive, dose-related trend (Figure 5 and Additional file 2: Figure S2). In addition, the concentration of Zn in liver and kidney was much higher than in the spleen and brain. In spleen and brain tissue, the Zn concentration showed no dose-related response compared with the vehicle control, even in the high-dose group.

### Excretion of nanoparticles

**Figure 6:**
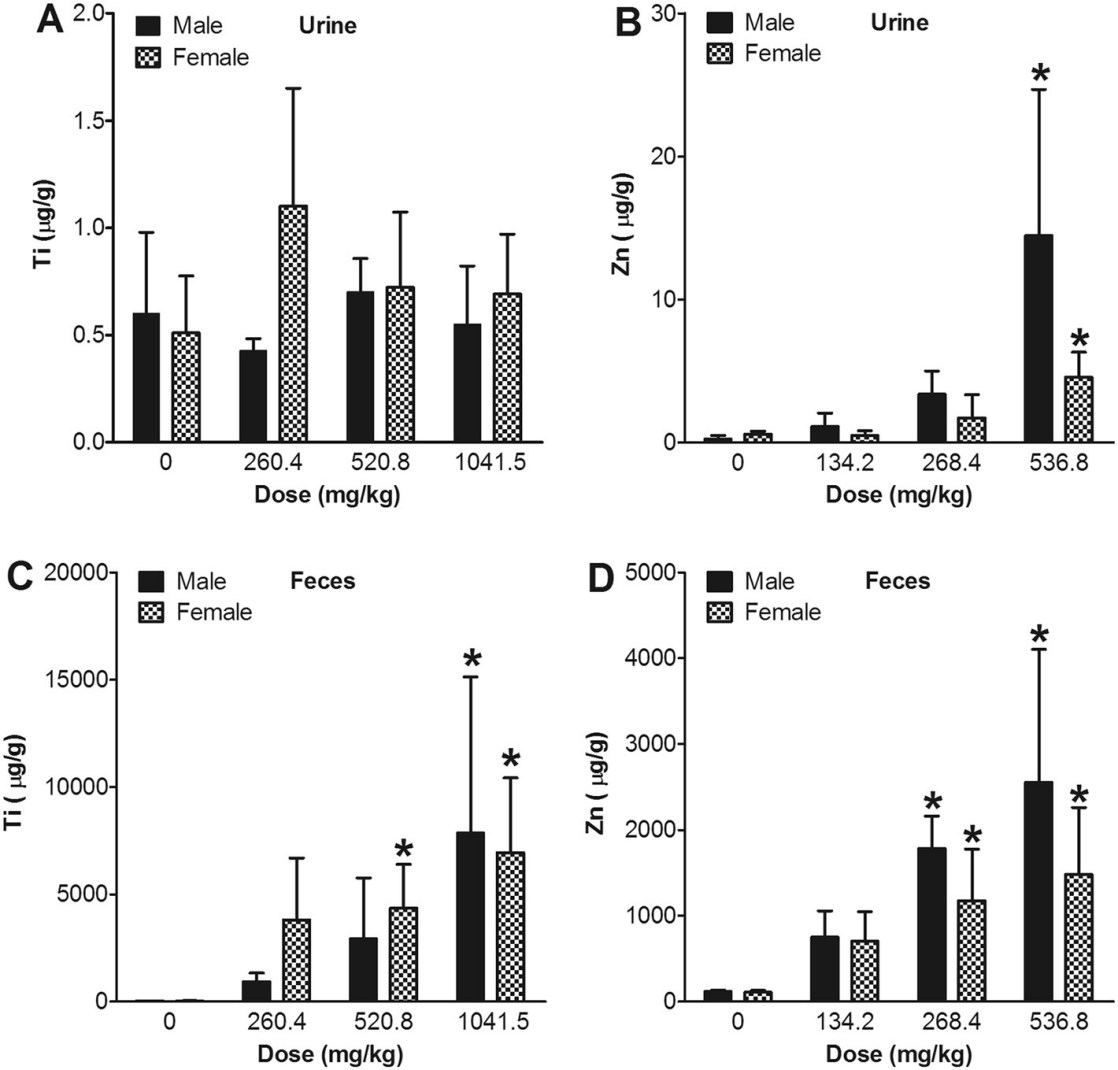
**Excretion of nanoparticles after 13 weeks of consecutive oral administration.** The concentrations of Ti in urine (**A**) and feces (**C**) were measured using an ICP-MS for TiO_2_ treatment groups. The concentrations of Zn in urine (**B**) and feces (**D**) were measured using an ICP-MS for ZnO treatment groups. Values are mean ± S.D. and *n* = 11. Significance versus vehicle control: ^*^p < 0.05.

The urine concentration of Ti in the TiO_2_-treatment groups showed no significant differences compared with the control group, which was consistent with the tissue distribution patterns (Figure 6A). In contrast, the concentration of Zn in the urine of ZnO-treatment groups was significantly increased in the middle- and high-dose groups and showed positive trend dose-responses (Figure 6C). Ti or Zn concentrations in the feces were very high compared to concentrations in the urine or tissues, with clear dose responses. These results suggested that most of the nanoparticles were not absorbed from the gastrointestinal lumen (Figures 6B and 6D).

## Discussion

TiO_2_ and ZnO particles are widely used as ingredients and food supplements [3,6,7]. As nanotechnology develops, these traditional materials have also been applied in nanoparticulate form. However, nanoparticles interact with biological systems through different mechanisms of action than bulk chemicals [13]. Nanoparticles are considered to be more highly absorbed into the respiratory, skin, and gastrointestinal systems than micron-sized particles because of their unique physicochemical properties, such as their size and surface modifications [14]. For example, instillation of 5 nm fluorescent nanoparticles shows faster translocation to other organs than 27 nm nanoparticles [15]. In addition, a recent study showed that only nanoparticles were translocated into the circulation system when administered into the lung; their absorption rate varied, depending on their surface properties [16]. The uptake of particles into skin cells was observed to be size dependent [17]. When different sizes of polystyrene particles were administered orally to rats, the absorption of 50–100 nm polystyrene nanoparticles was about 250-fold higher than absorption of larger microparticles (500 nm, 1, and 10 μm) [18,19]. Oral administration of different sizes of colloidal gold particles to mice showed size-dependent absorption [20]. Regarding surface modification, positively charged particles show better absorption than neutral or negatively charged particles [21]. However, in a recent study using gold nanoparticles, negatively charged particles showed higher absorption rates than positively charged particles [22]. Therefore, no general rule can be made about absorption from these results and further studies are needed on the impact of surface modification on gastrointestinal absorption. Studying the kinetics of nanoparticles is an important issue in nanotechnology. Compared with inhalation or skin exposure, the oral intake of nanoparticles in food-related settings has the potential for wide exposure of the public to higher doses and more frequent ingestion [1,2]. This study focused on the kinetics of the nano-particulate forms of TiO_2_ and ZnO as representative food-related nanomaterials.

Both particle types showed very low absorption although the exact absorption rate could not be calculated. The concentration of Ti in the blood samples of rats treated with TiO_2_ for 13 weeks was 0.4–0.5 μg/g and the differences between the values of the control and high-dose groups were less than 0.1 μg/g. Considering that the dose for the high-dose group was 1041.5 mg/kg body weight, TiO_2_ nanoparticles might have an extremely low absorption rate, although additional experiments are needed to confirm this. In contrast, the Zn concentration in the blood of rats in the ZnO-treatment groups showed an almost 10-fold higher dose and a steeper dose-response curve than values for animals in the Ti groups. The higher concentration of Zn in blood compared to Ti might be because of the biopersistence of nanoparticles. TiO_2_ and ZnO nanoparticles have different durability under acidic conditions, although both are minimally destructive in basic or physiological conditions. In our previous study, ZnO nanoparticles showed rapid dissolution in acidic conditions (pH 5.5) [12,23]. Because the pH of the gastric fluid is between 1.5 and 2.0, ZnO nanoparticles administered orally must dissolve in the stomach, with Zn ions then absorbed to enter into systemic circulation. In contrast, the low absorption rate of TiO_2_ might be because of their low solubility. The low absorption after oral administration of nanoparticles is consistent with the micron-sized biocompatible TiO_2_ particles [24]. Low absorption would be favorable from a risk perspective, especially in non-nutritional applications of nanoparticles such as in food preparation, handling, storage, and prevention of microbial growth. However, even with marginal absorption, some absorbed nanoparticles might pose a risk based on their toxic potential.

TiO_2_ distribution to the liver, spleen, kidney, and brain was also minimal. No dose-response relationship was seen, meaning that the TiO_2_ particles were not significantly distributed. This low distribution was due to the minimal absorption rate. This result was in contrast with a previous study that observed systemic translocation of TiO_2_ nanoparticles through the gastrointestinal tract [3]. Therefore, further studies and advanced methods to detect nanoparticles in the tissues are warranted. However, ZnO nanoparticles showed significant levels of Zn in the tested organs, particularly in the liver and kidney, with steep dose-response curves. In a previous study, orally administered ZnO nanoparticles were mainly distributed to the liver and kidney within 72 h following administration [25,26]. When two different sizes of ZnO were administered, the particle size and gender of animals did not influence the tissue distribution pattern [25]. In addition, orally administered zinc-65 was mainly distributed in the liver, muscle, lung, kidneys, and bone [27].

Data on the excretion of the nanoparticles were consistent with the absorption and distribution patterns. The Ti concentration in urine showed no significant differences from the controls, while the Zn concentration in urine was significantly higher than the vehicle controls, with clear dose-responses. ZnO nanoparticles administered orally are excreted via the urine [25]. The main routes of elimination of nanoparticles are urine/kidney and bile/liver. Renal excretion was confirmed in several studies using a multi-walled carbon nanotube [28] and quantum dots [29]. However, studies using quantum dots [30,31] and gold nanoparticles [32] showed long-term accumulation in the organs and no or minimal excretion via urine. The number of nanoparticle elimination study is relatively limited and further studies are warranted.

## Conclusions

Zinc concentrations in blood, organs, and urine were higher than concentrations of titanium when nanoparticles were administered orally for 13 weeks. The kinetics of nanoparticles via oral administration might be influenced by interactions between the physicochemical properties of nanoparticles and the biological milieu with which they come into contact. The low absorption of TiO_2_ and ZnO nanoparticles might be favorable, especially for non-nutritional applications. More thorough instigations regarding the impact of physicochemical properties on the kinetics of nanoparticles are warranted.

## Methods

### Nanoparticles and physicochemical characterization

Powder-form TiO_2_ nanoparticles were obtained from ABC Nanotech Co., Ltd. (Daejeon, Korea). Well-dispersed ZnO nanoparticles at 20 wt% in distilled water (DW) were obtained from Nanostructured and Amorphous Materials, Inc. (TX, USA). Primary particle sizes and morphology were measured using scanning electron microscopy (S-3500 N, Hitachi Science Systems, Ltd., Japan) and transmission electron microscopy (LEO-912AB Omega, LEO, Japan). The hydrodynamic size and zeta potential of nanoparticles in DW were measured using a DelsaNano (Beckman Coulter, Inc., CA, USA) according to the manufacturer’s instructions. Briefly, the optimal concentration of TiO_2_ and ZnO for measurement was screened by serial dilution under following conditions: temperature 25°C, refractive index 1.33, scattering angle 165°, laser wavelength 632.8 nm. The zeta potential of nanoparticles was measured under acidic conditions (pH 2.0) to simulate acidic gastric conditions, and under basic conditions (pH 8.0) to simulate the small intestine. Levels of endotoxin contamination were measured using an Endpoint Chromogenic Limulus Amebocyte Lysate assay (Cambrex, MD, USA).

### Durability of nanoparticles in biological conditions

TiO_2_ and ZnO nanoparticles were incubated with acidic gastric fluid (AGF, pH 1.5–2.0) or under basic conditions (pH 7.4). AGF was prepared according to a previously described method [33]. Briefly, 2.0 g NaCl (Sigma-Aldrich, St. Louis Mo. USA) and 3.2 g pepsin (Sigma-Aldrich) were dissolved in 1 L of DW and the pH was adjusted to 1.5 using 2 N HCl (Sigma-Aldrich). DW at pH 7.4 was used for the basic solution. Nanoparticles at 5 mg/mL were incubated in solution for up to 24 h. The degree of ionization was evaluated at 24 h post-incubation using inductively coupled plasma-mass spectrometry (ICP-MS). Briefly, at 24 h after incubation in solution, nanoparticle-free supernatants were collected by three rounds of centrifugation at 15000 × *g* for 30 min. The concentrations of zinc or titanium were measured using ICP-MS (X7; Thermo Elemental, Winsford, UK) as previously described method [34]. Gross images were taken using a digital camera (Nikon Corp., Tokyo, Japan).

### Animal handling and protocols

Six-week-old specific-pathogen free Sprague-Dawley rats were obtained from OrientBio Ltd. (Seongnam, Korea) and acclimated for seven days after arrival at the study facility. Rodents were housed in an animal room at a controlled temperature (21–24ºC), humidity (45–60%), and light cycle (12 h light/dark). Autoclaved water and gamma-irradiated rodent diets (LabDiet 5002, PMI nutrition, Richmond, USA) were provided *ad libitum*. The concentration of zinc in the diet was 74 ± 7.1 ppm as provided by the manufacturer (PMI Nutrition, Richmond, USA) and was not detectable in water provided by the Office of Waterworks (Seoul Metropolitan Government, Seoul, Korea). The concentrations of titanium in the diet and water were not known. All animal experiments were performed according to the Organization for Economic Co-operation and Development test guideline (TG 408) and the Good Laboratory Practices for toxicity test guidance (Notification No. 2005-60, Oct. 21, 2005) issued by the Korea Food and Drug Administration. Animal experiment protocols were approved by the Animal Care and Use Committee of Seoul National University Hospital, Korea.

### Dose-range finding study for 13 weeks of repeated oral nanoparticle administration

A single-dose acute toxicity study and a 14-day repeated toxicity study of the oral route were performed. For the 14-day study, TiO_2_ was administered orally at 520.8, 1041.5, and 2083 mg/kg/day, and ZnO was administered at 536.8, 1073.5, and 2147 mg/kg/day. Survival, clinical signs, body weight change, hematological change, serum biochemical change, organ weight, and necropsy findings were evaluated for MTD endpoints. There were no treatment-related findings in the TiO_2_ study; the highest dose over 13 weeks was selected as 1041.5 mg/kg/day for studying long-term exposure. For ZnO, clinical signs (erection of fur, depression, abdominal inflation, and diarrhea) and a more than 10% reduction of body weight were observed at doses over 1073.5 mg/kg in both sexes. Several hematological parameters and the level of alkaline phosphatase were significantly differed than in the vehicle control (data not shown). Based on these data, the maximum tolerated dose (MTD) was determined to be 1041.5 for TiO_2_ and 536.8 for ZnO nanoparticles.

### 13-week repeated oral administration of nanoparticles

Eleven rats were assigned to each of four treatment groups: vehicle control (DW), ZnO nanoparticles at 134.2, 268.4, and 536.8 mg/kg/day, and TiO_2_ nanoparticles at 260.4, 520.8, and 1041.5 mg/kg/day. All nanoparticles were suspended in DW at each working concentration and orally administered at 10 ml/kg body weight according to the Handbook of Toxicology, second edition [35]. Administration volume was adjusted based on body weight measured each week. Each group was treated with same concentration of nanoparticles for the entire 13 weeks. Oral administration was done 7 days per week without any anesthesia or starvation. Animals were weighed weekly and observed daily for clinical signs and mortality. Animals were sacrificed and dissected one day after final gavage.

### Absorption, distribution, and excretion of nanoparticles

Blood was taken via the abdominal vein at necropsy followed by isoflurane euthanization. To evaluate tissue distribution, tissue samples from the liver, spleen, kidney, and brain were obtained and weighed. Nanoparticles excretion was tested in urine and feces. To collect urine and feces, five animals from each group were randomly assigned to a metabolic cage immediately after gavage, and urine and feces samples were collected for 24 h. All samples were analyzed for elemental zinc and titanium to represent ZnO and TiO_2_ concentrations. ICP-MS was used to analyze zinc and titanium concentrations in samples as previously described [34]. The detection limit of ICP-MS was 0.1–1 ng/L for Ti and 0.1–10 ng/L for Zn.

### Statistical analysis

ICP-MS data were analyzed using one-way ANOVA (JMP ver. 4.0, NC, USA). Animal data were analyzed using TDMS ver 4.0 (KFDA, Osong, Korea). When statistically significant differences were indicated (*p* < 0.05), a Dunnett’s *t*-test was employed for comparisons between the control and treatment groups.

## Competing interests

The authors declare that they have no competing financial interests.

## Authors’ contributions

WSC, JHC and SHS provided key intellectual input in the conception and design of these studies, performed experiment, and aided in the writing of this manuscript. BCK provided expertise for kinetics data and contributed to the writing of the manuscript. JKL and JJ performed dissolution studies and contributed to the writing of the manuscript. All authors read and approved the final manuscript.

## Supplementary Material

Additional file 1: Table S1 Body weight changes of male SD rats treated with TiO2 nanoparticles for 13 weeks. **Table S2.** Body weight changes of female SD rats treated with TiO2 nanoparticles for 13 weeks. **Table S3.** Body weight changes of male SD rats treated with ZnO nanoparticles for 13 weeks. **Table S4.** Body weight changes of female SD rats treated with ZnO nanoparticles for 13 weeks. Click here for file

Additional file 2: Figure S1 Total concentrations of Ti per organs after 13 weeks of consecutive oral administration of TiO_2_ nanoparticles. Values are mean ± S.D. and *n* = 11. Significance versus vehicle control: ^*^p < 0.05. **Figure S2.** Total concentrations of Zn per organs after 13 weeks of consecutive oral administration of ZnO nanoparticles. Values are mean ± S.D. and *n* = 11. Significance versus vehicle control: ^*^p < 0.05. Click here for file
