# Supplementary material for: Comparative absorption, distribution, and excretion of titanium dioxide and zinc oxide nanoparticles after repeated oral administration
Source: Part Fibre Toxicol. 2013 Mar 26;10:9. doi: 10.1186/1743-8977-10-9 (PMC3616827; doi:10.1186/1743-8977-10-9)
Supplement: Additional file 1: Table S1 — Body weight changes of male SD rats treated with TiO2 nanoparticles for 13 weeks. Table S2. Body weight changes of female SD rats treated with TiO2 nanoparticles for 13 weeks. Table S3. Body weight changes of male SD rats treated with ZnO nanoparticles for 13 weeks. Table S4. Body weight changes of female SD rats treated with ZnO nanoparticles for 13 weeks. [file 1743-8977-10-9-S1.pdf]

Table S1. Body weight changes of male SD rats treated with TiO_2 nanoparticles for 13 weeks.

		0 mg/10ml/kg	260.37 mg/10ml/kg	520.75 mg/10ml/kg	1,041.5 mg/10ml/kg
Week 1	Day 1	260.6±16.1	255.4±13.9	259.0±10.6	259.9±12.9
Week 2	Day 1	321.6±22.0	313.6±21.5	317.4±14.9	322.2±19.1
Week 3	Day 1	368.0±27.8	361.6±28.4	363.0±22.7	367.3±27.6
Week 4	Day 1	409.6±35.0	403.7±37.8	404.2±26.2	411.3±33.1
Week 5	Day 1	438.3±42.0	435.2±45.2	424.2±26.9	442.3±34.7
Week 6	Day 1	467.0±45.2	466.5±46.6	455.1±33.7	476.0±41.2
Week 7	Day 1	487.4±55.8	480.6±46.2	475.1±35.5	497.7±47.0
Week 8	Day 1	511.4±55.2	500.3±53.5	498.5±40.3	520.4±51.3
Week 9	Day 1	534.5±66.8	518.1±58.3	519.6±43.6	536.2±51.4
Week 10	Day 1	551.3±71.3	533.1±62.6	534.5±45.8	551.4±54.6
Week 11	Day 1	563.5±75.6	551.7±65.9	548.2±47.6	563.7±56.5
Week 12	Day 1	576.4±76.9	566.1±69.1	565.9±50.0	581.8±59.6
Week 13	Day 1	585.2±81.0	579.7±69.0	575.6±50.0	595.1±61.4

Table S2. Body weight changes of female SD rats treated with TiO_2 nanoparticles for 13 weeks.

		0 mg/10ml/kg	260.37 mg/10ml/kg	520.75 mg/10ml/kg	1,041.5 mg/10ml/kg
Week 1	Day 1	170.0±9.8	171.3±11.8	172.4±9.7	168.2±10.5
Week 2	Day 1	198.5±14.2	198.3±14.0	198.5±12.3	197.0±12.5
Week 3	Day 1	224.7±16.9	224.9±14.7	223.2±14.1	223.4±15.5
Week 4	Day 1	245.4±21.0	243.1±14.8	243.3±18.3	237.1±16.4
Week 5	Day 1	257.8±22.8	255.3±16.2	256.8±18.7	248.2±19.3
Week 6	Day 1	273.0±26.0	270.9±19.8	265.7±17.7	262.7±20.8
Week 7	Day 1	281.2±26.2	278.0±19.1	277.8±19.3	268.0±24.2
Week 8	Day 1	283.0±28.0	285.4±17.4	280.5±19.7	273.8±22.4
Week 9	Day 1	288.7±28.4	292.2±20.7	289.9±19.7	279.6±26.4
Week 10	Day 1	300.3±27.9	302.0±23.8	296.8±22.0	288.0±26.0
Week 11	Day 1	300.8±29.5	304.0±23.3	300.8±23.9	290.4±25.8
Week 12	Day 1	311.8±28.0	311.4±22.1	306.8±25.6	297.6±29.0
Week 13	Day 1	316.3±31.7	315.2±23.3	313.2±28.7	301.7±27.5

Table S3. Body weight changes of male SD rats treated with ZnO nanoparticles for 13 weeks.

		0 mg/10ml/kg	67.1 mg/10ml/kg	134.2 mg/10ml/kg	268.4 mg/10ml/kg	536.8 mg/10ml/kg
Week 1	Day 1	260.8±9.8	261.1±9.8	265.1±13.3	265.0±13.9	259.1±10.2
Week 2	Day 7	316.6±16.5	317.5±16.6	323.0±20.8	320.9±17.3	304.2±16.6
Week 3	Day 7	358.5±21.3	355.6±26.5	365.3±29.4	361.5±21.1	346.6±16.8
Week 4	Day 7	393.5±27.6	395.8±28.6	403.3±35.3	396.6±25.1	365.3±26.9
Week 5	Day 7	426.7±35.2	424.2±33.7	434.6±36.2	427.5±26.9	388.5±33.5*
Week 6	Day 7	454.2±34.9	451.3±36.7	456.1±32.8	457.2±27.5	413.5±39.3*
Week 7	Day 7	474.7±38.1	471.1±36.0	478.7±38.9	478.2±28.5	436.0±35.8
Week 8	Day 7	496.7±43.5	495.2±38.0	500.4±41.5	497.8±29.6	456.1±36.3
Week 9	Day 7	514.4±45.1	513.0±40.2	519.8±42.3	515.8±29.2	468.3±39.3*
Week 10	Day 7	534.6±49.5	533.4±43.3	546.7±47.2	531.9±27.6	482.5±49.4*
Week 11	Day 7	550.0±53.2	550.4±44.2	566.7±49.7	550.4±28.6	485.6±59.6*
Week 12	Day 7	555.4±56.8	556.1±46.9	571.0±48.1	551.3±34.4	494.6±52.9*
Week 13	Day 7	561.2±56.9	561.5±48.5	570.8±48.7	559.7±34.1	504.8±54.0*

Values are mean ± S.D. and $n = 11$. Significance versus vehicle control: $^{*}p < 0.05$.

Table S4. Body weight changes of female SD rats treated with ZnO nanoparticles for 13 weeks.

		0 mg/10ml/kg	67.1 mg/10ml/kg	134.2 mg/10ml/kg	268.4 mg/10ml/kg	536.8 mg/10ml/kg
Week 1	Day 1	179.8±9.6	180.2±8.6	181.0±7.8	181.4±10.0	179.2±8.2
Week 2	Day 7	207.7±11.1	209.2±11.2	207.0±10.9	210.3±13.5	207.9±8.4
Week 3	Day 7	223.9±11.4	224.0±14.4	221.1±11.9	229.1±19.1	226.8±11.1
Week 4	Day 7	241.7±13.5	245.0±16.2	239.8±14.1	247.4±17.9	242.6±12.9
Week 5	Day 7	251.3±18.7	254.4±18.5	250.6±17.1	259.1±21.7	259.6±15.9
Week 6	Day 7	260.2±18.1	264.3±17.6	259.4±15.8	264.2±20.9	264.4±15.5
Week 7	Day 7	265.3±16.7	269.0±18.0	261.4±14.8	269.0±20.9	269.4±15.4
Week 8	Day 7	270.2±17.0	277.5±20.5	268.1±19.6	273.8±21.7	274.7±15.0
Week 9	Day 7	278.1±17.3	284.3±21.3	273.3±22.9	283.4±26.1	280.4±17.6
Week 10	Day 7	287.5±17.0	289.7±23.8	282.9±23.3	291.8±24.1	285.1±19.8
Week 11	Day 7	294.4±17.6	300.2±20.9	288.1±23.7	298.9±29.8	293.2±15.7
Week 12	Day 7	295.5±16.2	302.4±22.1	290.1±20.8	300.4±30.9	296.5±15.8
Week 13	Day 7	301.5±19.1	306.9±24.3	291.0±23.8	301.5±30.0	295.3±15.7

Values are mean ± S.D. and $n = 11$. Significance versus vehicle control: $^{*}p < 0.05$.
