# Supplementary material for: Comparative absorption, distribution, and excretion of titanium dioxide and zinc oxide nanoparticles after repeated oral administration
Source: Part Fibre Toxicol. 2013 Mar 26;10:9. doi: 10.1186/1743-8977-10-9 (PMC3616827; doi:10.1186/1743-8977-10-9)
Supplement: Additional file 2: Figure S1 — Total concentrations of Ti per organs after 13 weeks of consecutive oral administration of TiO2 nanoparticles. Values are mean ± S.D. and n = 11. Significance versus vehicle control: *p < 0.05. Figure S2. Total concentrations of Zn per organs after 13 weeks of consecutive oral administration of ZnO nanoparticles. Values are mean ± S.D. and n = 11. Significance versus vehicle control: *p < 0.05. [file 1743-8977-10-9-S2.docx]

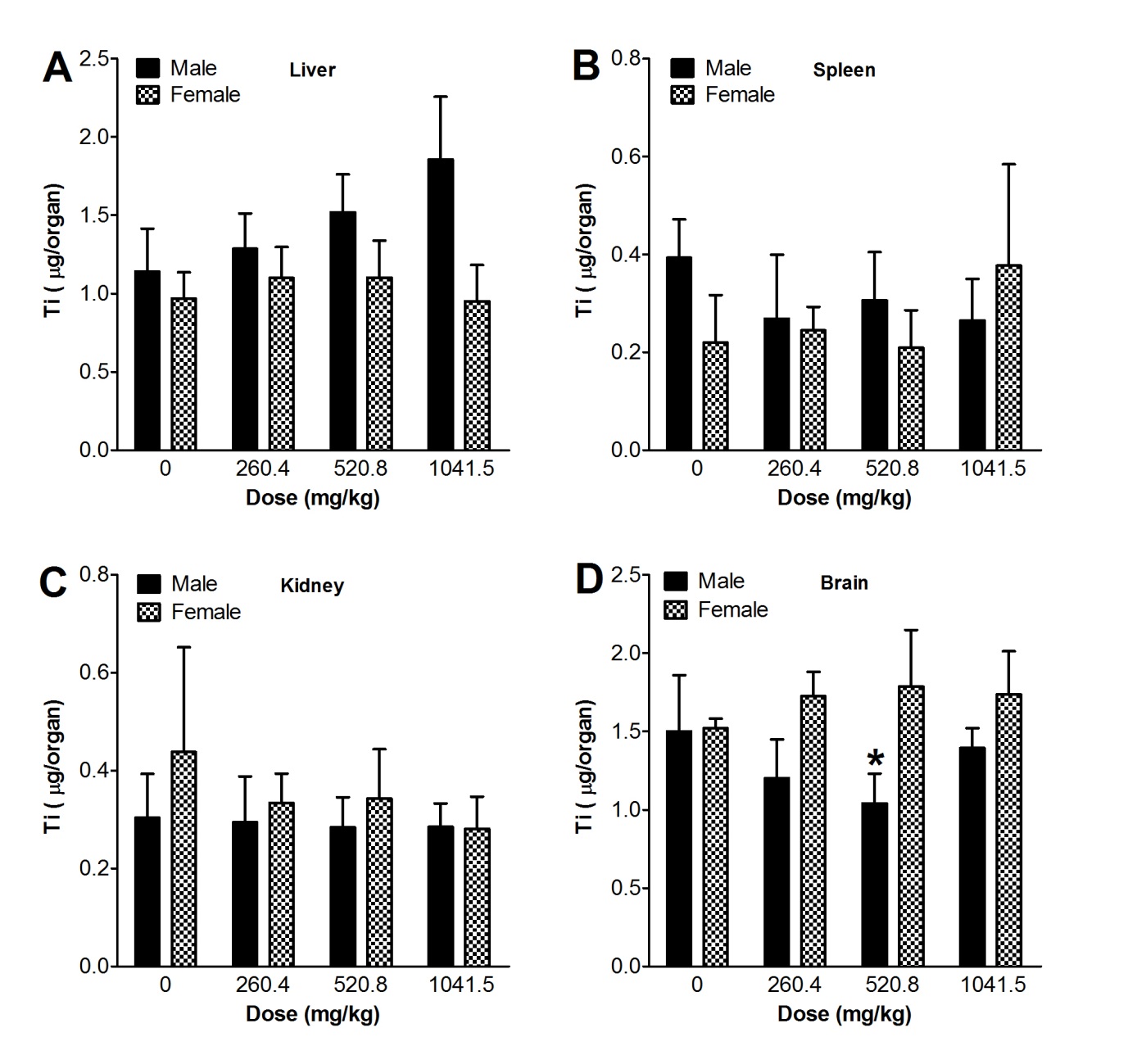


Figure S1. Total concentrations of Ti per organs after 13 weeks of consecutive oral administration of TiO_2_ nanoparticles. Values are mean ± S.D. and *n* = 11. Significance versus vehicle control: ^*^ *p* < 0.05.


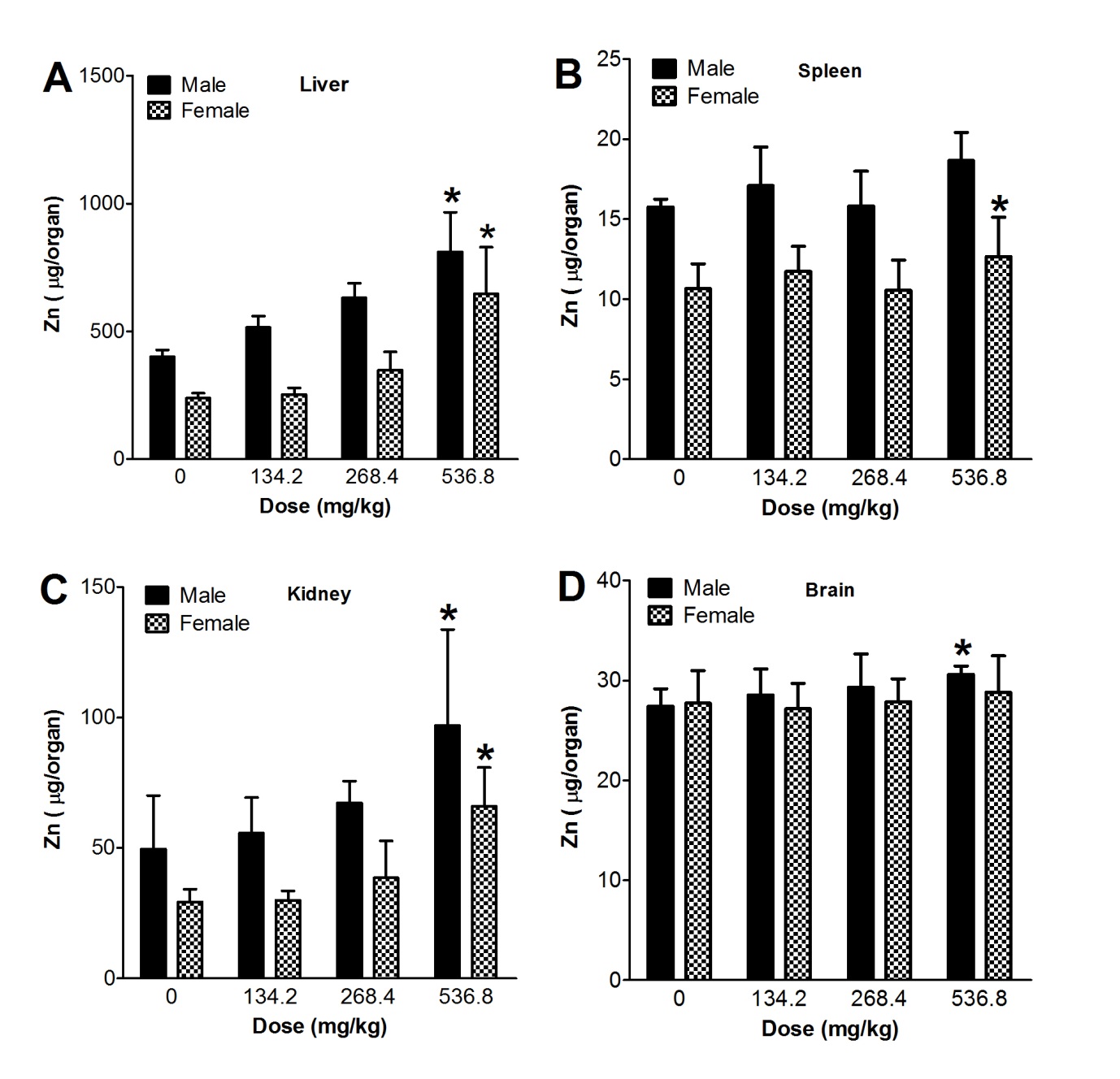


Figure S2. Total concentrations of Zn per organs after 13 weeks of consecutive oral administration of ZnO nanoparticles. Values are mean ± S.D. and *n* = 11. Significance versus vehicle control: ^*^ *p* < 0.05.
